# Association of serum and salivary dipeptidyl peptidase-4 (DPP-4) with oral cancerous and precancerous lesions; an observational diagnostic accuracy study

**DOI:** 10.1186/s12903-024-04939-7

**Published:** 2024-10-10

**Authors:** Nayroz Abdel Fattah Tarrad, Olfat Gamil Shaker, Maha Abdelkawy, Sandy Hassan

**Affiliations:** 1 Oral Medicine and Periodontology Department, Faculty of Dentistry, Fayoum University, Fayoum, Egypt; 2 Medical Biochemistry and Molecular Biology Department, Faculty of Medicine, Cairo University, Cairo, Egypt; 3 Oral Medicine and Periodontology Department, Faculty of Dentistry, Beni-Suef University, Beni-Suef, Egypt; 4 Oral Medicine and Periodontology Department, Faculty of Dentistry, Fayoum & Ahram-Candian Universities, Fayoum, Cairo Egypt

**Keywords:** Oral cancer, Saliva, Serum, Dipeptidyl peptidase, Oral potentially malignant lesions

## Abstract

**Background:**

Enhancing the prognosis and treatment outcomes of oral cancer relies heavily on its early detection. This study aims to establish a connection between serum and salivary dipeptidyl peptidase-4 (DPP-4) levels and oral squamous cell carcinoma (OSCC), comparing them with oral potentially malignant lesions (OPMLs) and control subjects and validating salivary DPP-4 as a diagnostic marker for the early detection of oral cancer.

**Methodology:**

Forty-five systemically healthy individuals were categorized into three groups: Group I consisted of 15 patients diagnosed with OSCC, Group II comprised 15 patients with OPMLs (including leukoplakia and oral lichen planus), and Group III included 15 participants without any oral mucosal lesions. Serum and whole unstimulated salivary samples were collected from all participants to assess DPP-4 levels using an enzyme-linked immunosorbent assay (ELISA) kit. Receiver operating characteristic (ROC) analysis was conducted to determine the area under the curve (AUC), sensitivity, specificity, and diagnostic accuracy of DPP-4.

**Results:**

Both serum and salivary DPP-4 levels were highest in the healthy group, followed by OPMLs, and lowest in the OSCC group, with statistically significant differences observed. ROC analysis demonstrated excellent diagnostic accuracy of salivary DPP-4 in distinguishing OSCC from healthy individuals, OPMLs from healthy individuals, and OSCC from OPMLs, with accuracies of 100%, 100%, and 96.67%, respectively. Salivary DPP-4 levels also exhibited a statistically significant correlation with OSCC grades.

**Conclusions:**

DPP-4 appears to play a protective, anti-oncogenic role in maintaining oral tissue health. The remarkable diagnostic accuracy of both serum and salivary DPP-4 in discriminating between OSCC, OPMLs, and healthy controls suggests its potential utility as a well-established marker for early oral cancer diagnosis. Salivary DPP-4, being non-invasive, could serve as a convenient chair-side diagnostic tool for the early detection of OSCC.

**Clinical trial registration:**

The study was retrospectively registered on clinicaltrial.gov with NCT06087042, date of registration (first posted date): 17/10/2023

## Background

Oral cancer (OC), ranking as the 6th most prevalent malignant tumor globally, exhibits a staggering 50% mortality rate within five years, with over 90% of cases attributed to oral squamous cell carcinoma (OSCC). Despite advancements, OC still carries a bleak prognosis, with survival rates exceeding 80% only if diagnosed at early stages (I and II) [1, 2]. Unfortunately, nearly half of OC cases are diagnosed in late stages (III and IV) due to the asymptomatic nature during initial phases, leading to delayed medical intervention [3]. Early detection holds the key to improving survival rates and mitigating treatment-related challenges such as swallowing, eating, and speaking limitations [4].

Oral potentially malignant lesions (OPMLs) encompass precursors to OSCC, including mucosal abnormalities like leukoplakia/erythroplakia, oral submucous fibrosis, and oral lichen planus [5, 6]. The lack of awareness among patients regarding risk factors and symptoms, coupled with inadequate preventive measures and limited early detection by healthcare providers, contribute to the late-stage diagnosis of OC [7, 8]. Hence, early identification and management of OPMLs are crucial in enhancing survival rates and reducing mortality.

While clinical examination, biopsy, and histological analysis remain the gold standard for OC and OPML diagnosis [9], recent research focuses on identifying effective clinical and molecular diagnostic techniques for early detection [10]. Dipeptidyl peptidase-4 (DPP-4/CD26), a cell surface glycoprotein involved in various biological functions ranging from immune regulation to glucose metabolism, also plays a role in carcinogenesis, either suppressing or activating tumors relying on the tumor microenvironment [11, 12]. Its soluble form (sCD26/sDPP4) present in body fluids, particularly serum/plasma, makes it a promising biomarker [13, 14]. Although altered expression of DPP-4 has been observed in various tumor types, including ovarian, thyroid, osteosarcoma, and colorectal cancers where increased levels were shown [15–18], its role in OSCC and OPMLs remains underexplored and to the best of our knowledge neither its serum nor salivary level was previously assessed regarding both lesions. The present study aims to assess the levels of DPP-4 in serum and saliva of individuals with both lesions to explore its potential as a diagnostic biomarker for early oral cancer detection, distinguishing it from OPMLs and healthy subjects.

## Methods

### Study population

In this prospective observational study, a total of 45 consecutive systemically healthy participants, spanning both genders and aged between 30 and 65 years, were enrolled. Participants were stratified based on the presence or absence of oral lesions and the type of oral lesions into three distinct groups: Group I: Comprised patients diagnosed with oral squamous cell carcinoma (*n* = 15), representing the malignant group. Group II: Encompassed patients diagnosed with potentially malignant lesions such as leukoplakia, and erosive/atrophic oral lichen planus (*n* = 15), serving as the premalignant group. Group III: Consisted of individuals exhibiting no oral mucosal lesions upon clinical examination (*n* = 15), serving as the healthy control group.

This study received ethical approval from the research ethics committee of the institutional review board of the Faculty of Oral and Dental Medicine at Ahram Canadian University (Approval number: IRB00012891#77). Additionally, the study was registered on clinicaltrial.gov (NCT06087042). Participants were consecutively selected from patients attending the outpatient clinics of the Department of Oral Medicine, Diagnosis, and Periodontology between August 2023 and October 2023. Prior to their inclusion in the study, all participants were provided with detailed information about the study’s objectives and procedures. Written consent was obtained from each participant before their enrollment.

### Inclusion criteria

1. Both genders with age range from 30 to 65 years old.
2. Systemically healthy participants.
3. Subjects having oral lesions diagnosed with only OSCC (for group I) and leukoplakia or atrophic/erosive OLP (for group II).
4. Nonsmoker / Non-Alcoholic.

### Exclusion criteria

1. Patients receiving any current medication (including contraceptive pills).
2. Pregnant or lactating females.
3. Subjects refusing to participate or sign the consent.

### Clinical examination

All participants underwent comprehensive oral diagnosis and were documented on diagnostic charts, followed by a thorough clinical examination. Medical evaluations were conducted on both patients and healthy individuals using the modified Cornell Medical Index [19]. Diagnosis of patients in Group I and II was initially established based on clinical findings and later confirmed through histopathological assessment. Histopathologic examination also determined the grade of dysplastic changes in biopsy specimens of oral potentially malignant lesions (OPMLs) if present [20]. Surgical incisional double wedge biopsies, approximately 2 mm in depth, were performed to obtain biopsy specimens from lesions [21]. These specimens were then sent to the pathology lab for histopathological assessment and confirmation of diagnosis after being coded with serial numbers. Oral lichen planus (OLP) lesions were classified into atrophic and erosive types according to Andreasen’s classification [22], which categorizes OLP lesions into six clinical forms (plaque, papular, bullous, atrophic, erosive, reticular). As OLP is among the most commonly faced OPMLs clinically [23], in addition red lesions of OLP are considered among the risk factors increasing its malignant transformation [24], accordingly atrophic and erosive forms were included in group II representing one of the OPMLs in this study. Leukoplakia lesions were classified as either homogenous or non-homogenous (speckled). Clinical data regarding lesions, including site, size, grade, stage, metastasis, and lymph node involvement in malignant patients, were recorded. Additionally, data on type, dysplastic grade, and presence of extraoral lesions in premalignant lesions were documented, along with ulcer and pain scores [25] for OLP using a visual analogue scale ranging from 0 to 10. Serum and whole unstimulated salivary samples were collected from all participants, including patients and controls, to evaluate and compare the level of DPP-4 across different groups using an ELISA kit.

### Whole unstimulated salivary samples collection

Unstimulated saliva samples were obtained in the morning using standardized methods as described by Navazesh [26]. Participants were instructed to refrain from smoking, eating, or drinking for approximately thirty minutes prior to sample collection. Collection involved instructing the individual to swallow and then tilt their head forward to expectorate saliva into a sterile tube. Subsequently, the samples were coded with serial numbers and stored at -20 °C until analysis.

### Serum samples collection

In sterile conditions and using plain tubes, 5 ml peripheral venous blood samples were obtained from all participants via standard venipuncture. These samples were then transported to the biochemistry laboratory where they underwent centrifugation and filtration to clarify the supernatant, which was subsequently stored at -20ºC for DPP-4 detection. Each sample was assigned a unique serial number for identification purposes.

The analysis of the ROC curve was conducted to assess the diagnostic significance of serum and salivary DPP-4 levels in distinguishing OSCC from OPMLs and healthy participants.

### Quantification of DPP-4 in serum & whole unstimulated saliva

The Enzyme-Linked Immunosorbent Assay (ELISA) kit used in this study was provided by Bioassay Technology Laboratory, with Cat No: E6631Hu (Shanghai, China).

### Reagents provided with the kit

Standard Solution (4.8U/L), Pre-coated ELIZA Plate, Standard Diluent, Streptavidin-HRP, Stop Solution, Substrate solution A, Substrate solution B, Wash Buffer concentration (25x), Biotinylated Human DPP4 Antibody.

### Assay procedure

(1). All reagents, standard solutions and samples were prepared as instructed. The assay was performed at room temperature. (2). 50 µl standard was added to standard well. (3). 40 µl sample was added to sample wells and then 10 µl anti-DPP4 antibody was added to sample wells, followed by 50 µl streptavidin-HRP was added to sample wells and added standard wells (not blank control well). Mixing well was done. The plate was covered with a sealer. Incubation was done for 60 min at 37 °C. (4). The sealer was removed, and the plate was washed 5 times with wash buffer. The plate was blotted onto paper towels or other absorbent material. (5). 50 µl substrate solution A was added to each well and then 50 µl substrate solution B was also added to each well. Incubation was done for the plate followed by covering the plate with a new sealer for 10 min at 37 °C in the dark. (6). 50 µl Stop Solution was added to each well, the blue color was changed into yellow immediately. (7). The optical density value of each well was determined immediately using a microplate reader set to 450 nm within 10 min after adding the stop solution.

The ELISA plate was pre-coated with Human DPP-4 antibody. DPP-4 present in the sample was added, binding to the antibodies coated on the wells. Subsequently, biotinylated Human DPP-4 Antibody was added, binding to DPP-4 in the sample. Streptavidin-HRP was then added, binding to the biotinylated DPP-4 antibody. During a washing step, any unbound Streptavidin-HRP was removed. Substrate solution was added, leading to color development in proportion to the amount of Human DPP-4 present. The reaction was halted by the addition of acidic stop solution, and absorbance was measured at 450 nm.

### Sample size calculation

This power analysis used DPP4 levels as the primary outcome. Based upon the results of Javidroozi M. et al. [27], the mean (*SD*) values of DPP4 levels were 3.87 (1.5) and 5.69 (1.47) µg/mL for head & neck cancer and healthy groups, respectively. Using alpha (α) level of (5%), β level of 0.8 (Power = 80%); the effect size (d) was 1.23 and the minimum estimated sample size was 12 subjects per group. G*Power version 3.1.9.2 was used to perform the sample size calculation.

### Statistical analysis

Categorical data were expressed as frequencies and percentages and were assessed using the chi-square test. Numerical data were presented as mean values with standard deviation (SD) and were tested for normality using Shapiro-Wilk’s test. As the data were normally distributed, they were analyzed using one-way ANOVA followed by Tukey’s post hoc test. Diagnostic accuracy was evaluated using ROC curve analysis, with cutoff values determined based on the highest Youden index. ROC curves were compared using the z-test. Correlations were assessed using Spearman’s rank order correlation coefficient. A significance level of *p* < 0.05 was applied to all tests. Statistical analysis was conducted using R statistical analysis software version 4.3.1 for Windows (R Core Team, 2023).

## Results

The study was conducted on 45 cases (i.e., 15 cases per group). There were 7 males and 8 females in the malignant and premalignant groups, while in the control group there were 5 males and 10 females. The mean age of the cases in the malignant group was (53.27 ± 7.27) years, in the premalignant group it was (46.07 ± 8.90) years while in the control group it was (47.87 ± 12.40) years. There was no significant difference between tested groups regarding gender (*p* = 0.695) and age (*p* = 0.247).

Clinical data for malignant and premalignant groups are presented in Tables 1 and 2 respectively. Site, size grade, stage, lymph node affection and metastasis are among the clinical criteria of malignant group. Type, grade of dysplasia and presence of extraoral lesions in addition to pain and ulcer scores for OLP patients are registered in the clinical features of the premalignant group.

**Table 1:** Clinical data for malignant group

Parameter		Value
** Site [n (%)] **	***Maxilla***	4 (26.7%)
	***Hard palate***	2 (13.3%)
	***Mandible***	2 (13.3%)
	***Tongue***	5 (33.3%)
	***Floor of mouth***	1 (6.7%)
	***Lip***	1 (6.7%)
** Grade [n (%)] **	***(I)***	1 (6.7%)
	***(II)***	9 (60.0%)
	***(III)***	5 (33.3%)
** Stage [n (%)] **	***(I)***	1 (6.67%)
	***(II)***	5 (33.33%)
	***(III)***	8 (53.33%)
	***(IV)***	1 (6.67%)
** Lymph nodes affection [n (%)] **	***No***	12 (80.0%)
	***Yes***	3 (20.0%)
** Metastasis [n (%)] **	***No***	14 (93.3%)
	***Yes***	1 (6.7%)
Size (cm ^3^ ) (Mean ± SD)		26.87 ± 16.47

**Table 2:** Clinical data for premalignant group

Parameter		Value
***Type of premalignant lesion*** **[n (%)]**	***Oral Lichen planus***	8 (53.3%)
	***Leukoplakia***	7 (46.7%)
***Grade of dysplasia*** **[n (%)]**	***No***	5 (33.3%)
	***Low***	8 (53.3%)
	***High***	2 (13.3%)
***Extraoral lesions*** **[n (%)]**	***No***	14 (93.3%)
	***Yes***	1 (6.7%)
***Pain score*** **(** ***Mean ± SD*** **)**		6.38 ± 3.62
***Ulcer score*** **(** ***Mean ± SD*** **)**		3.50 ± 1.07

Results of intergroup comparisons and summary statistics for the tested biochemical parameter in various samples are presented in Table 3. For both serum and salivary levels of DPP-4, there was a significant difference between all tested groups, with the control group having the highest value followed by the premalignant group and then the malignant group that showed the lowest value. All pairwise comparisons were statistically significant (*p* < 0.001).

**Table 3:** Intergroup comparisons of serum and salivary DPP-4 levels

Parameter	(Mean ± SD) (ug/ml)			Statistic	*p*-value
	Malignant	Premalignant	Control		
**Serum DPP-4**	5.41 ± 0.92^C^	7.80 ± 0.87^B^	9.23 ± 1.47^A^	44.53	< 0.001*
**Salivary DPP-4**	28.78 ± 4.14^C^	42.13 ± 5.08^B^	56.23 ± 3.05^A^	162.32	< 0.001*

Values with different superscript letters within the same horizontal row are significantly different, *significant (*p* < 0.05)

Results of the correlations between both levels of the biomarker (serum, saliva) in each group are presented in Table 4. For all groups, there was a strong positive correlation between the biomarker levels in both salivary and serum samples that was statistically significant (rs > 0.8, *p* < 0.001).

**Table 4:** Correlations between serum DPP-4 and salivary DPP-4 in different groups

Group	Correlation coefficient (95% CI)	*p*-value
**Malignant**	0.898 (0.714:0.966)	< 0.001*
**Premalignant**	0.994 (0.981:0.998)	< 0.001*
**Control**	0.984 (0.951:0.995)	< 0.001*

*Significant (*p* < 0.05)

**Fig. 1:**
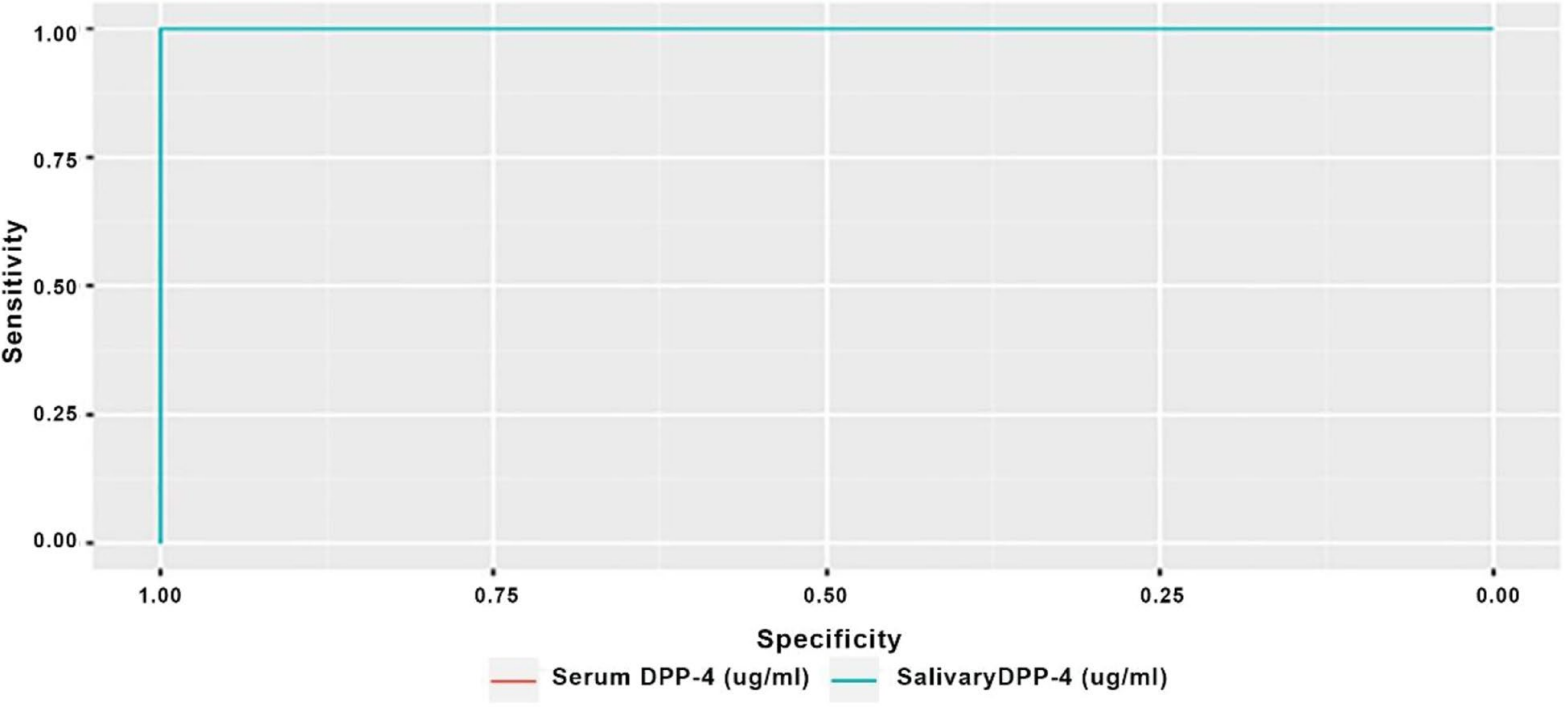
ROC curve differentiating malignant group from control group

**Fig. 2:**
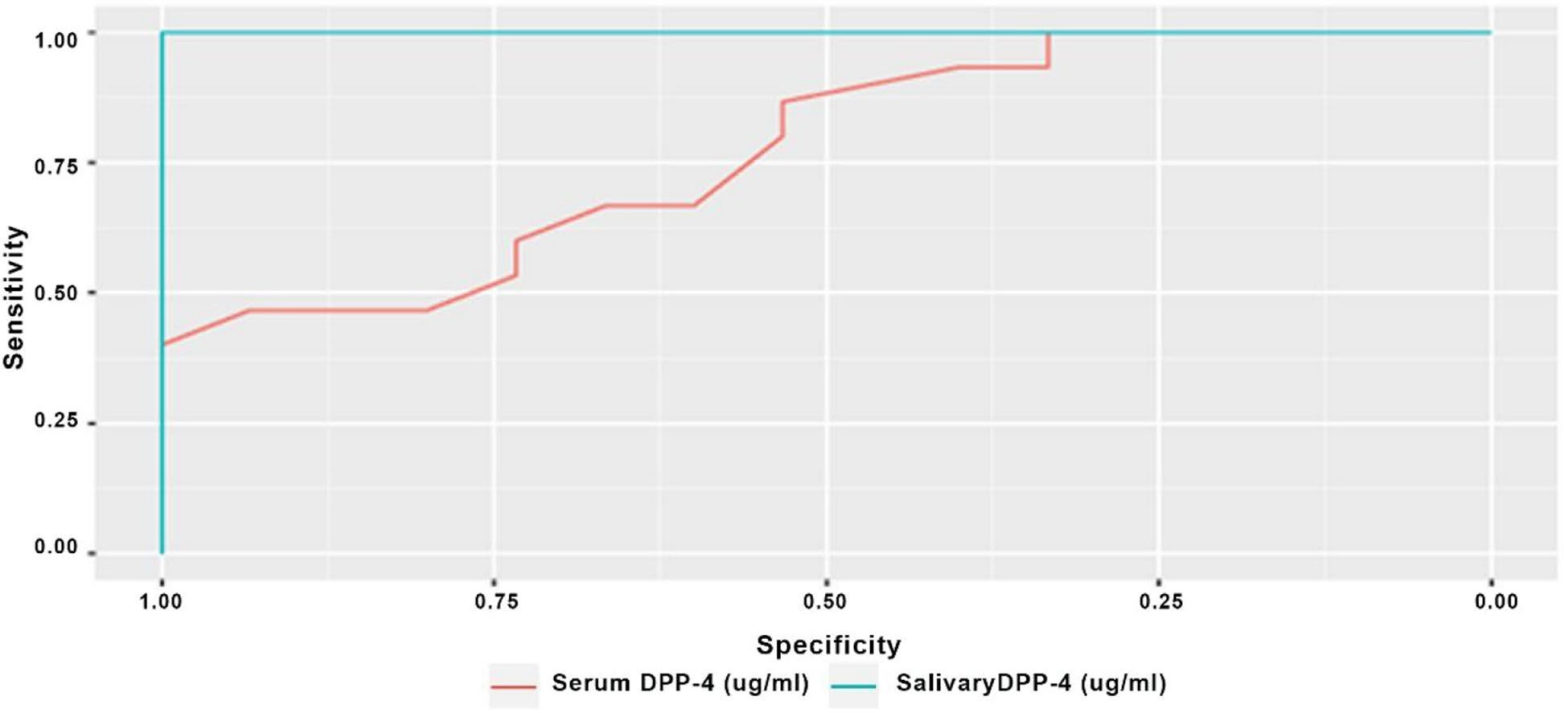
ROC curve differentiating premalignant group from control group

**Fig. 3:**
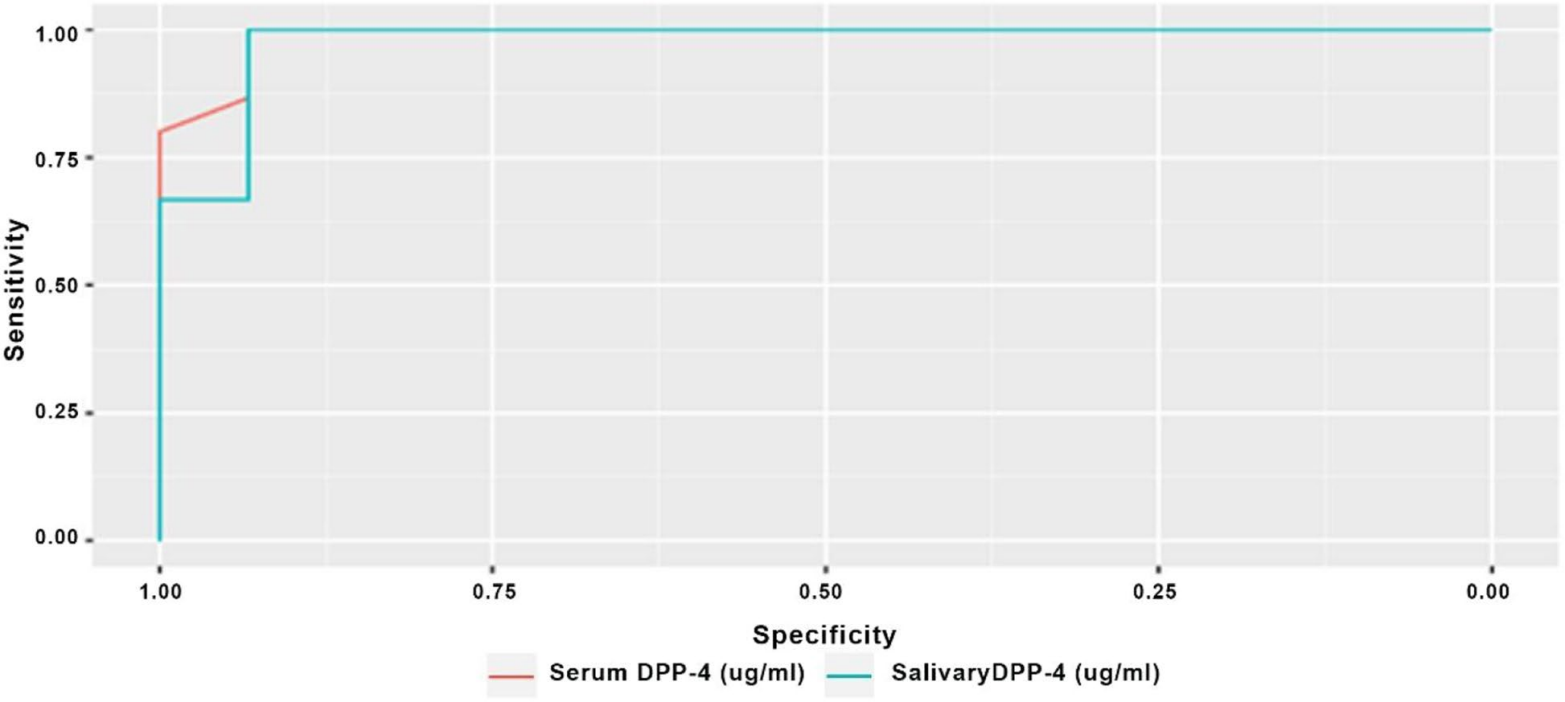
ROC curve differentiating malignant and premalignant groups

Results of ROC curve analyses are presented in Table 5 and in Figs 1, 2 and 3. Results showed that, accuracy of both serum and salivary DPP-4 had an outstanding diagnostic ability in discriminating the malignant group from both the control and the premalignant cases (AUC > 0.9) with the difference between both levels being not statistically significant (*p* > 0.05). While for the premalignant group, salivary DPP-4 had significantly higher discriminating ability against healthy control in comparison to serum DPP-4 (*p* = 0.008).

**Table 5:** Diagnostic accuracy of serum and salivary DPP-4 to differentiate different included groups

Differentiating	Parameter	Sensitivity (95%CI)	Specificity (95%CI)	Accuracy (95%CI)	Cut off point	NPV (95%CI)	PPV (95%CI)	AUC (95%CI)	AUC difference (95%CI)	*p*-value
**OSCC from control group**	**Serum DPP-4**	100.00% (100.00%:100.00%)	100.00% (100.00%:100.00%)	100.00% (100.00%:100.00%)	<=7.09	100.00% (100.00%:100.00%)	100.00% (100.00%:100.00%)	1.000 (1.000:1.000)	0.000 (0.000:0.000)	1
	**Salivary DPP-4**	100.00% (100.00%:100.00%)	100.00% (100.00%:100.00%)	100.00% (100.00%:100.00%)	<=43.87	100.00% (100.00%:100.00%)	100.00% (100.00%:100.00%)	1.000 (1.000:1.000)		
**OPMLs from control group**	**Serum DPP-4**	73.33% (33.33%:100.00%)	80.00% (40.00%:100.00%)	76.67% (63.33%:86.67%)	<=9.28	75.00% (60.00%:100.00%)	81.25% (60.00%:100.00%)	0.776 (0.609:0.942)	0.224 (-0.391:-0.058)	0.008*
	**Salivary DPP-4**	100.00% (100.00%:100.00%)	100.00% (100.00%:100.00%)	100.00% (100.00%:100.00%)	<=50.40	100.00% (100.00%:100.00%)	100.00% (100.00%:100.00%)	1.000 (1.000:1.000)		
**OSCC from OPMLs group**	**Serum DPP-4**	100.00% (86.67%:100.00%)	93.33% (80.00%:100.00%)	96.67% (90.00%:100.00%)	<=6.57	100.00% (88.24%:100.00%)	93.75% (83.33%:100.00%)	0.989 (0.964:1.000)	0.011 (-0.014:0.036)	0.383
	**Salivary DPP-4**	100.00% (100.00%:100.00%)	93.33% (80.00%:100.00%)	96.67% (90.00%:100.00%)	<=33.77	100.00% (100.00%:100.00%)	93.75% (83.33%:100.00%)	0.978 (0.931:1.000)		

*Significant (*p* < 0.05)

Associations and correlations between levels of serum/salivary DPP-4 and different clinical parameters in malignant and premalignant groups are presented in Tables 6, 7, 8 and 9. Results showed that for the malignant group there was a significant association between serum DPP-4 and grading, with grade (III) cases having a significantly lower value than other grades (*p* < 0.001). There was also a significant association between grading and salivary DPP-4 with grade (I) cases having the highest value, followed by grade (II) cases and with grade (III) cases having the lowest value and with all post hoc pairwise comparisons being statistically significant (*p* < 0.001). For premalignant group, cases with OLP had significantly lower values of both markers in comparison to leukoplakia cases (*p* < 0.001). Other correlations and associations were not statistically significant (*p* > 0.05).

**Table 6:** Associations of DPP-4 with clinical data in malignant group

Marker	Parameter		(Mean ± SD) (ug/ml)	Test statistic	*p*-value
***Serum DPP-4***	**Grade**	**(I)**	6.80 ± 0.00^A^	19.70	< 0.001*
		**(II)**	5.83 ± 0.55^A^		
		**(III)**	4.36 ± 0.29^B^		
	**Stage**	**(I)**	4.20 ± 0.00^A^	2.25	0.139
		**(II)**	6.00 ± 0.47^A^		
		**(III)**	5.32 ± 0.96^A^		
		**(IV)**	4.30 ± 0.00^A^		
	**Lymph nodes affection**	**No**	5.62 ± 0.88	2.04	0.062
		**Yes**	4.53 ± 0.49		
	**Metastasis**	**No**	5.43 ± 0.95	0.33	0.743
		**Yes**	5.10 ± 0.00		
***Salivary DPP-4***	**Grade**	**(I)**	38.10 ± 0.00^A^	37.05	< 0.001*
		**(II)**	30.23 ± 2.01^B^		
		**(III)**	24.30 ± 0.55^C^		
	**Stage**	**(I)**	24.20 ± 0.00^A^	1.23	0.345
		**(II)**	31.00 ± 2.48^A^		
		**(III)**	28.42 ± 4.70^A^		
		**(IV)**	25.10 ± 0.00^A^		
	**Lymph nodes affection**	**No**	29.48 ± 4.26	1.34	0.204
		**Yes**	26.00 ± 2.38		
	**Metastasis**	**No**	28.79 ± 4.29	0.02	0.985
		**Yes**	28.70 ± 0.00		

Values with different superscript letters within the same vertical column and parameter are significantly different, *significant (*p* < 0.05)

**Table 7:** Correlations of DPP-4 with lesion size in malignant group

Variables	Correlation coefficient (95% CI)	*p*-value
***Serum DPP-4- size***	0.031 (-0.489:0.535)	0.912
***Salivary DPP-4- size***	-0.154 (-0.618:0.389)	0.583

*Significant (*p* < 0.05)

**Table 8:** Associations of DPP-4 with clinical data in premalignant group

Marker	Parameter		(Mean ± SD) (ug/ml)	Test statistic	*p*-value
***Serum DPP-4***	**Type of premalignant lesion**	**Oral Lichen planus**	7.09 ± 0.41	7.56	< 0.001*
		**Leukoplakia**	8.61 ± 0.37		
	**Grade of dysplasia**	**No**	7.38 ± 0.80	1.07	0.375
		**Low**	7.92 ± 0.87		
		**High**	8.35 ± 1.06		
	**Extraoral lesions**	**No**	7.88 ± 0.85	1.34	0.203
		**Yes**	6.70 ± 0.00		
***Salivary DPP-4***	**Type of premalignant lesion**	**Lichen planus**	37.77 ± 1.66	10.72	< 0.001*
		**Leukoplakia**	47.10 ± 1.70		
	**Grade of dysplasia**	**No**	40.14 ± 4.69	0.60	0.562
		**Low**	42.83 ± 5.27		
		**High**	44.30 ± 6.79		
	**Extraoral lesions**	**No**	42.57 ± 4.96	1.30	0.216
		**Yes**	35.90 ± 0.00		

*Significant (*p* < 0.05)

**Table 9:** Correlations of DPP-4 with pain/ulcer scores in premalignant group

Variables	Correlation coefficient (95% CI)	*p*-value
***Serum DPP-4- pain score***	0.538 (-0.269:0.901)	0.169
***Salivary DPP-4- pain score***	0.496 (-0.321:0.890)	0.212
***Serum DPP-4- ulcer score***	-0.351 (-0.846:0.470)	0.394
***Salivary DPP-4- ulcer score***	-0.332 (-0.840:0.486)	0.422

## Discussion

Typically, the prognosis for the most severe tumors worsens when they reach advanced stages or are located in inaccessible areas [28]. Detecting oral malignancies early, coupled with proactive diagnosis, leads to improved prognosis, higher survival rates, and reduced treatment complications [29]. Among oral lichen planus (OLP) variants, the atrophic and ulcerative types pose the highest risk of malignant transformation (MT), with a 25.8-fold increased likelihood of progressing to oral squamous cell carcinoma (OSCC) compared to hyperplastic OLP [30]. Additionally, oral leukoplakia exhibits a significant risk of MT into oral cancer, previously estimated at 9.8% [31].

Although DPP-4 is typically regarded as a pro-oncogene, it has been observed to play an anti-oncogenic role in certain tumors such as glioma and prostate cancer [14]. Previous cohort studies on individuals with type 2 diabetes have linked treatment with DPP-4 inhibitors (DPP-4i) to various cancers including thyroid tumors, pancreatic cancer, and cholangiocarcinoma [32–34]. Our current findings regarding oral squamous cell carcinoma (OSCC) highlight the anti-oncogenic role of DPP-4. Both serum and salivary levels of DPP-4 were highest in the healthy group, followed by the oral potentially malignant lesions (OPMLs) group, and lowest in the OSCC group, indicating a significant role of DPP-4 in maintaining oral mucosal health. These results align with a previous study examining plasma DPP-4 levels across different cancer types, including head and neck cancers, which found significantly lower DPP-4 levels in cancer patients compared to healthy individuals [27]. Additionally, the decreased expression of DPP-4 observed in our OSCC group is consistent with findings in patients with hepatocellular carcinoma reported by Yu et al. [35].

A recent systematic review from 2021 suggests that DPP-4 levels may serve as a promising biomarker for monitoring inflammatory bowel disease activity [36]. Furthermore, CD26/DPP-4 expression has demonstrated prognostic and diagnostic potential in hematological tumors [37]. Our findings also support the notion that serum and salivary DPP-4 could serve as diagnostic biomarkers for the diseases included in our study. According to the results of our ROC analysis, both serum and salivary DPP-4 exhibited excellent sensitivity, specificity, and diagnostic accuracy in distinguishing OSCC from control subjects and OSCC from OPMLs. Interestingly, salivary DPP-4 displayed superior diagnostic performance compared to serum DPP-4 in differentiating OPMLs from control subjects. This suggests for the 1st time, to the best of our knowledge, that salivary DPP-4 may offer reliable and more advantageous diagnostic potential for both OSCC and OPMLs due to its less invasive collection method, lower risk of infection, and simpler sampling process compared to serum DPP-4. Similar observations have been made in other tumors, where DPP-4 expression specificity has been validated as a robust biomarker for chronic myeloid leukemia (CML) in peripheral blood. It has been proven to be a cost-effective, patient friendly and efficient flow cytometry procedure, aiding in the rapid exclusion of CML [38].

Our findings revealed a statistically significant strong positive correlation between serum and salivary DPP-4 levels across all groups. Furthermore, the correlation analysis between clinical data and either serum or salivary DPP-4 levels indicated that salivary DPP-4 exhibited a better significant correlation with various grades of OSCC compared to serum DPP-4. Specifically, salivary DPP-4 demonstrated significance across different grades of OSCC, while serum DPP-4 showed significance only between grade III and the other grades. Notably, the lowest levels of both serum and salivary DPP-4 were observed in grade III, whereas the highest levels were found in grade I. These results suggest that DPP-4, particularly in its salivary form, may effectively differentiate between different grades of OSCC.

In contrast, our study did not find any correlation between the stage of OSCC and either serum or salivary DPP-4 levels. This aligns with an earlier investigation that examined serum DPP-4 levels in OSCC patients and healthy individuals [39] but differs from a more recent study conducted by Javidroozi et al. [27]. In their study, they observed a significant decrease in plasma DPP-4 levels in stage III cancer compared to stages I, II, and IV, considering the overall TNM staging of various cancers included in their analysis. The variance in findings could be attributed to their inclusion of different cancer types alongside head and neck cancer in their correlation between DPP-4 levels and cancer stage, while our study focused solely on OSCC. However, similar to our findings, their study did not reveal any significant differences in DPP-4 levels concerning tumor size or lymph node involvement.

Extensive research has explored the correlation between various potential markers and the grade of dysplasia among different OPMLs [40]. Intriguingly, when examining the relationship between serum/salivary DPP-4 levels and dysplastic grades of OPMLs, it was observed that the high dysplastic grade exhibited higher DPP-4 levels compared to low-grade dysplasia or the absence of dysplasia, although this difference lacked statistical significance. A systematic review examining the malignant transformation (MT) rates of different OPMLs, including OLP and oral leukoplakia, found that OLP had an MT rate of 1.4%, whereas leukoplakia showed a much higher MT rate of 9.5% [41]. This suggests that DPP-4 levels may be associated with the risk of malignant transformation in various OPMLs, indicating that the elevation in DPP-4 levels as dysplastic changes increase could stem from the normal mucosal cells’ attempt to compensate for and control the dysplastic mucosal alterations occurring in each OPML.

Reduced levels of DPP-4 have been associated with numerous diseases characterized by inflammatory processes. A prior study [42] on rheumatoid arthritis (RA) patients demonstrated lower DPP-4 values in subjects with RA, along with a decrease in proinflammatory cytokines associated with increased DPP-4 expression. Additionally, literature indicates that patients with inflammatory bowel disease also exhibit decreased circulatory levels of CD26/DPP-4 [43]. Our findings corroborate these earlier observations, as OLP displayed significantly lower levels of DPP-4 in both saliva and serum compared to healthy controls. Furthermore, leukoplakia is well known to have higher malignant transformation than OLP [41] which was notably reflected on its higher DPP-4 levels than in OLP. This could also be attributed to the inflammatory nature of OLP, which plays a prominent role in its pathogenesis, whereas leukoplakia lacks such inflammatory processes.

Previous studies showed that DPP-4 activities in serum of OSCC patients when registered at different intervals of treatment revealed a dynamic change in its activities reflecting the clinical status of patients where DPP-4 values were elevated with tumor regression and reduced with cancer progression. Moreover, these elevated activities were associated with fair prognosis while poor prognosis is linked to lower levels [39]. It was also previously shown that patients suffering from head and neck cancer and had higher plasma DPP-4 level had better survival [27]. This could add evidence and support our results regarding the role of elevated DPP-4 levels registered in the healthy control group in maintaining healthy state of the oral mucosa.

DPP-4 inhibitors have been demonstrated to boost the anti-cancer effects of immunotherapy. Yet, a recent study stated that DPP-4 inhibitors considerably worsen the results for colorectal cancer patients who have undergone curative surgery. They explained that this could be due to the acceleration of Epithelial-Mesenchymal Transition and the establishment of an immune microenvironment that is conducive to tumor growth [44].

A recent research finding revealed that the DPP-4 activity in the serum is lower in samples of OSCC compared to normal ones. Interestingly, they found neither the mRNA level of DPP-4 in the tissue nor the activity of DPP-4 in the tissue showed any correlation with the activity of DPP-4 in the paired serum. They suggested that the circulating DPP-4 does not come from the tumor tissue of OSCC patients. However, it’s important to consider that the number of patients included in their study was relatively small and the used serum detection techniques and kits were different [45].

Despite the identification of several biomarkers in saliva, there is a pressing need to pinpoint those with superior reliability and diagnostic efficacy [46, 47]. Traditionally, serum or plasma has been the preferred bodily fluid for precise evaluation of biomarker levels in clinical investigations [48]. However, in our study, salivary DPP-4 exhibited comparable potential to reflect the condition of the oral mucosa in both health and disease. This could be attributed to its close contact and proximity to the oral mucosa, highlighting the notion that saliva may serve as a more suitable reservoir for various biomarkers related to oral mucosal lesions, particularly for DPP-4 in the cases of OSCC and OPMLs.

Taken together and owing to the strong positive correlation found between serum and salivary DPP-4 in various groups and their excellent diagnostic accuracy, sensitivity and specificity, salivary DPP-4 could be used as a non-invasive efficient tool to early detect oral malignancy which is inexpensive, easily collected and does not require any trained personal.

Limitations of our study include the relatively small sample size, underscoring the need for further investigations involving larger populations and the subdivision of each OPML into independent groups with more recommendation for OPMLs like leukoplakia and erythroplakia having higher MT than OLP. Not applying the clinical policy bulletin (CPB) in different clinical stages is another limitation of this study, suggesting other investigations that stratify the groups into initial versus advanced stages. Additionally, it is recommended to examine tissue samples from various OPMLs and OSCC to assess the presence of DPP-4 and compare it with other well-established tumor biomarkers. Furthermore, exploring the impact of different treatment modalities for both OPMLs and OSCC on salivary DPP-4 levels is essential to evaluate its prognostic value.

## Conclusions

DPP-4 appears to play a protective, anti-oncogenic role in maintaining oral tissue health. Both serum and salivary DPP-4 levels correlate well in malignant and premalignant lesions. The remarkable diagnostic accuracy of both serum and salivary DPP-4 in discriminating OSCC from OPMLs and healthy control could suggest its potential utility as a well-established marker for early oral cancer diagnosis. Salivary DPP-4, being non-invasive, could serve as a convenient chair-side diagnostic technique for early detection of OSCC.

## Data Availability

The datasets used and/or analyzed during the current study are available from the corresponding author on reasonable request.

## Abbreviations

OSCCs: Oral squamous cell carcinomas
OPMLs: Oral potentially malignant lesions
OLP: Oral lichen planus
DPP-4: Dipeptidyl peptidase -4
OC: Oral cancer
ROC: Receiver Operating Characteristic
ELISA: Enzyme linked immune-sorbent assay
MT: Malignant transformation
RA: Rheumatoid arthritis
CML: Chronic myeloid leukemia
CPB: Clinical policy bulletin
